# Beyond Strict Physics: Using Poiseuille’s Law as a Practical Framework to Optimize and Personalize Cementoplasty

**DOI:** 10.3390/jpm16010041

**Published:** 2026-01-08

**Authors:** Sylvain Grange, Rémi Grange, Vincent Habouzit, Maxime Pastor, Louis-Martin Boucher, Jean-Pierre Pelage, Natalia Gorelik, Nicolas Stacoffe

**Affiliations:** 1 Service de Radiologie, CHU Saint-Etienne Université Jean Monnet, Mines Saint-Etienne, INSERM, SAINBIOSE U1059, 42055 Saint-Etienne Cedex, France; 2 Department of Diagnostic Radiology, McGill University Health Centre (MUHC-Glen)-Royal Victoria Hospital, Montreal, QC H3A 1A1, Canadanatalia.gorelik@mail.mcgill.ca (N.G.); 3 Service de Radiologie, CHU Saint-Etienne, Université Jean Monnet, 42055 Saint-Etienne Cedex, France; remi.grange@chu-st-etienne.fr; 4 Department of Nuclear Medicine, Saint-Etienne University Hospital, University of Saint-Etienne, 42055 Saint-Etienne Cedex, France; vincent.habouzit@chu-st-etienne.fr; 5 Osteoarticular Medical Imaging Section, Department of Medical Imaging, Lapeyronie Hospital, 34295 Montpellier Cedex 5, France; 6 Department of Radiology, Groupement Hospitalier Sud, Hospice Civils de Lyon, 69495 Pierre-Bénite Cedex, France; nicolas.stacoffe@chu-lyon.fr

**Keywords:** bone cements, cementoplasty, vertebroplasty, injections, intralesional, viscosity, pressure, interventional radiology, personalized medicine

## Abstract

**Background/Objectives:** Poiseuille’s law describes the influence of radius, length, viscosity, and pressure on the flow of Newtonian fluids. Although bone cement is a non-Newtonian, shear-thinning, and polymerizing material that does not comply with this law in any predictive or quantitative sense, its qualitative principles may offer a didactic framework for understanding factors that affect injectability during cementoplasty. The objective of this Technical Note is to provide an educational and conceptual interpretation of Poiseuille’s law as it relates to trocar selection, cement behavior, and procedural planning. **Methods:** This work presents theoretical calculations based on the r^4^/L component of Poiseuille’s equation, using manufacturer-specified internal radii for commonly used trocars. Relative flow rates were computed as r^4^/L ratios normalized to a 13-gauge, 15 cm trocar. Conceptual viscosity profiles illustrate qualitative differences among cements over time. A representative, fully anonymized clinical example is provided to illustrate the integration of these conceptual principles into practice. No experimental measurements were performed. **Results:** Theoretical calculations show that trocar radius has the strongest influence on theoretical flow, with an exponential effect (r^4^), whereas increasing trocar length proportionally reduces flow. Conceptual viscosity curves demonstrate the rapid rise in viscosity during polymerization and highlight the importance of timing and cement selection. The clinical example illustrates how trocar choice, access planning, and cement viscosity are adapted to lesion morphology and cortical integrity. **Conclusions:** Poiseuille’s law cannot model or predict bone cement behavior and has no procedural or clinical validity in cementoplasty. Its use in this Technical Note is strictly educational, providing a qualitative framework to illustrate general relationships between equipment characteristics, viscosity evolution, and resistance during injection, without offering clinical guidance or implying any impact on procedural planning, safety, or outcomes.

## 1. Background/Objectives

Poiseuille’s law, also known as Hagen–Poiseuille’s law, is not usually emphasized in medical curricula, yet it provides an essential framework for understanding fluid dynamics in clinical practice [1]. The purpose of this Technical Note is strictly didactic: Poiseuille’s law is used only as a conceptual framework to illustrate qualitative relationships between radius, length, viscosity, and pressure, and not as a quantitative or predictive model of bone cement behavior. The law describes laminar flow of a Newtonian fluid through a cylindrical tube of constant radius and length, where flow rate is determined by four key variables: the pressure gradient, the tube radius, the tube length, and the fluid viscosity [2]. Although bone cement is widely used in interventional radiology for cementoplasty, it is not a Newtonian fluid [3]. Instead, bone cements exhibit time-dependent, shear-thinning, and polymerizing behavior that fundamentally precludes the predictive or quantitative application of Poiseuille’s law [4]. For this reason, Poiseuille’s law cannot be applied in a predictive or procedural manner to bone cement; in this manuscript, it is used solely as a conceptual and educational analogy to illustrate general qualitative relationships between trocar diameter, length, viscosity, and injection pressure. This conceptual approach does not guide procedural decision-making but simply provides a qualitative illustration of how equipment-related factors may relate to resistance during injection, while actual cement behavior is determined by lesion type, cortical integrity, bone microarchitecture, venous drainage patterns, and real-time viscosity changes. In recent years, vertebroplasty has become one of the most frequent minimally invasive interventions for painful vertebral lesions in many interventional radiology centers [5]. Beyond its technical success, vertebroplasty has been associated in many studies with pain reduction and improved mobility, although results have varied depending on indication, trial design, and imaging findings, particularly in the context of osteoporotic fractures, where randomized controlled data have reported heterogeneous outcomes. Nonetheless, several studies—particularly in metastatic spinal disease and selected osteoporotic fractures—have reported meaningful improvements in pain and mobility, although results remain dependent on indication and study design. The principles highlighted by Poiseuille’s law exemplify how interventional radiology aligns with precision medicine to improve safety and therapeutic efficacy. Beyond technical considerations, the therapeutic objective must also be defined according to the patient’s will, age, general condition, and both current and expected quality of life, making the decision to perform cementoplasty part of a truly personalized medical approach [6]. For example, the exponential relationship between tube radius and flow rate (r^4^ factor) highlights the importance of trocar gauge selection, while the role of length illustrates why shorter trocars reduce resistance and facilitate injection [2]. Cement leakage, which has been reported in approximately 20–40% of procedures depending on indication and imaging modality, is more frequently detected on CT than on fluoroscopy; most cases are clinically silent, although neurologic, vascular, or soft-tissue complications may rarely occur [7,8]. Lesion-specific factors such as cortical disruption, trabecular architecture, venous drainage patterns, and tumor cellularity significantly influence leakage risk and must guide both cement selection and injection strategy. Prevention relies on a combination of high-viscosity cement when appropriate, controlled and incremental pressurization, real-time imaging guidance, and procedural planning that anticipates pathways of least resistance [7].

Thus, while Poiseuille’s law does not apply *stricto sensu* to bone cement, its principles remain valuable to interventional radiologists as a heuristic model for personalizing cementoplasty [9,10], integrating physics with lesion biology and patient-specific anatomy to maximize safety and therapeutic efficacy.

**Figure 1:**
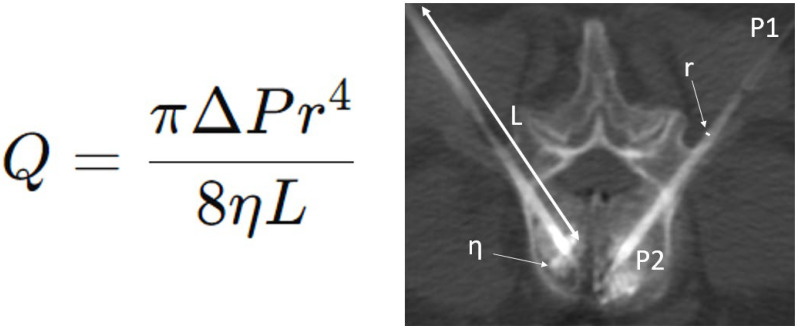
Poiseuille’s law equation (**left**) and application during a bipedicular L3 cementoplasty (**right**). Q = volume flow rate (m^3^/s); r = radius (m); L = length (m); η = fluid of dynamic viscosity (Pa.s); ΔP = pressure difference (Pa) between the two ends of the tube.

**Figure 2:**
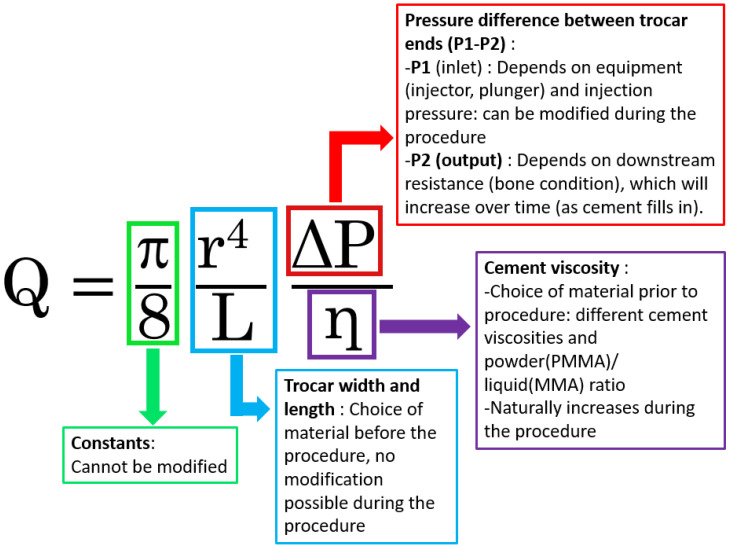
**Poiseuille’s law equation applied to cementoplasty.** The diagram illustrates how flow rate (Q) is influenced by trocar radius (r), trocar length (L), injection pressure difference (ΔP), and cement viscosity (η). PMMA is shown as an example of a biological cement, acknowledging its non-Newtonian behavior but highlighting the didactic value of Poiseuille’s law for understanding and optimizing cementoplasty.

For a tube of radius r (m), length L (m), and a fluid of dynamic viscosity η (Pa.s), Poiseuille’s law formulates the volume flow rate Q (m^3^/s) as a function of the pressure difference ΔP (Pa) between the two ends of the tube (Figure 1). To better understand this law and its underlying modifiable and non-modifiable factors, the operator can simplify the equation (Figure 2). This law equation is composed of several elements, including unmodifiable constants and modifiable factors that can influence the flow rate. The flow rate can be adjusted in equipment-related choices, such as the width and length of the trocar, as well as the cement type and composition. It can also be influenced by changing the cement injection pressure. In the following sections, each component of the equation is discussed in detail.

## 2. Materials and Methods

1. Theoretical framework and assumptions (Figure 1 and Figure 2)

Poiseuille’s law was used exclusively as a conceptual model to illustrate qualitative flow relationships. The equation used for illustrative calculations was as follows:

Q = (π·r^4^·ΔP)/(8·η·L),

where Q is the theoretical flow rate (m^3^/s); r is the inner radius of the trocar (m); L is the length (m); η is dynamic viscosity (Pa·s); and ΔP is the pressure gradient (Pa). Units were converted from millimeters to meters for radius values. No attempt was made to model bone cement behavior, which is non-Newtonian and time-dependent.

2. Computation of the “relative flow rate”

The relative flow rate values presented in Table 1 were calculated using the r^4^/L ratio, normalized to the 13 G–15 cm trocar as reference (set to 1.00). All calculations were performed using inner radius values expressed in meters and rounded to two decimals for readability. These values represent theoretical predictions and not experimental measurements.

**Figure 3:**
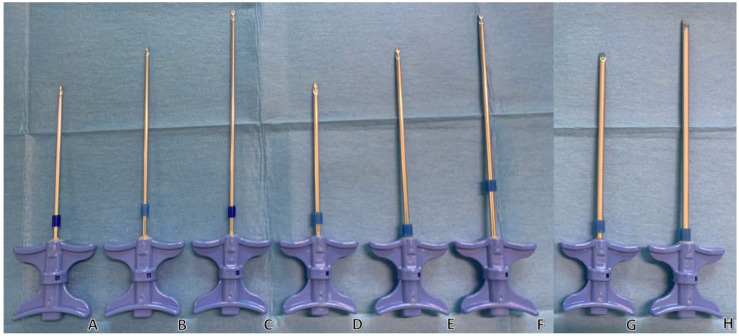
Examples of trocars with different lengths and diameters. (Thiebaud/Strim Health Care trocars, Thonon-les-Bains, France): t’AM (**A**–**F**) and t’OCR (**G**,**H**). Trocars are shown in three diameters: 13-gauge (**A**–**C**), 11-gauge (**D**–**F**) and 8-gauge (**G**,**H**); and in three lengths: 100 mm (**A**,**D**), 125 mm (**B**,**E**,**G**), and 150 mm (**C**,**F**,**H**) trocars. The wider and shorter the trocar, the easier the injection of PMMA cement.

3. Source of internal radius values (Figure 3)

Internal radius values for 8 G, 11 G, 13 G, and 18 G trocars were obtained from manufacturer specifications (Thiebaud/Strim Healthcare, Thonon-les-Bains, France). These values were used solely to illustrate conceptual flow differences.

**Figure 4:**
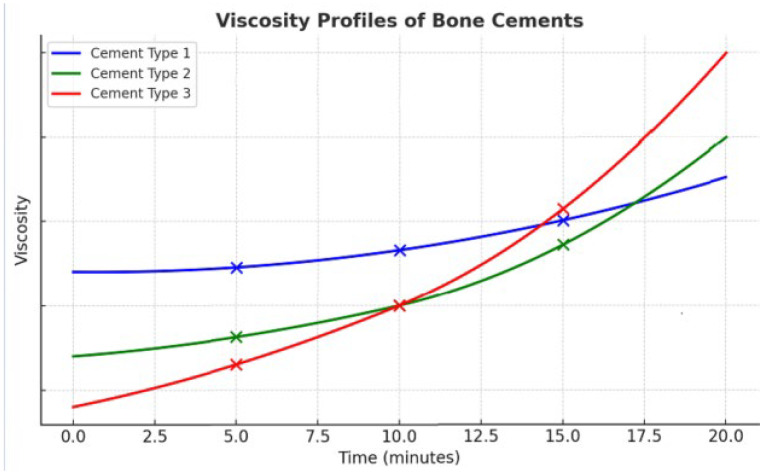
**Viscosity profiles of three bone cements over time.** The diagram highlights three measurement points (5, 10, and 15 min) to illustrate the differences in viscosity kinetics. At 5 min, Cement Type 1 (blue) shows the highest viscosity, Cement Type 2 (green) remains intermediate, and Cement Type 3 (red) is the most fluid, favoring easy injection. At 10 min, Cement Type 1 (blue) stays nearly stable, Cement Type 2 (green) begins to rise more noticeably, while Cement Type 3 (red) initiates a sharp upward trend (“knee effect”). At 15 min, Cement Type 3 (red) becomes the most viscous, Cement Type 2 (green) surpasses Cement Type 1 (blue), which, despite starting as the thickest, has now become the least viscous. These contrasting patterns underline the importance of cement selection during procedures: Cement Type 3 (red) offers high injectability at the beginning but rapidly hardens and becomes unsuitable beyond 10–12 min; Cement Type 1 (blue), though more viscous initially, remains stable and workable for a longer period, supporting extended injection; Cement Type 2 (green) provides an intermediate option, starting moderately fluid and then accelerating later, ensuring a balance between early and late usability. *The viscosity curves shown are illustrative and do not represent experimental measurements; they serve to demonstrate qualitatively how different cements may exhibit distinct viscosity kinetics over time.*

4. Viscosity curves (Figure 4)

The viscosity profiles shown in Figure 4 are conceptual illustrations based on qualitative trends described in the literature. They do not represent experimental measurements. Their purpose is to highlight the diversity of viscosity evolution patterns among commercially available cements.

5. Clinical example and ethical considerations

**Figure 5:**
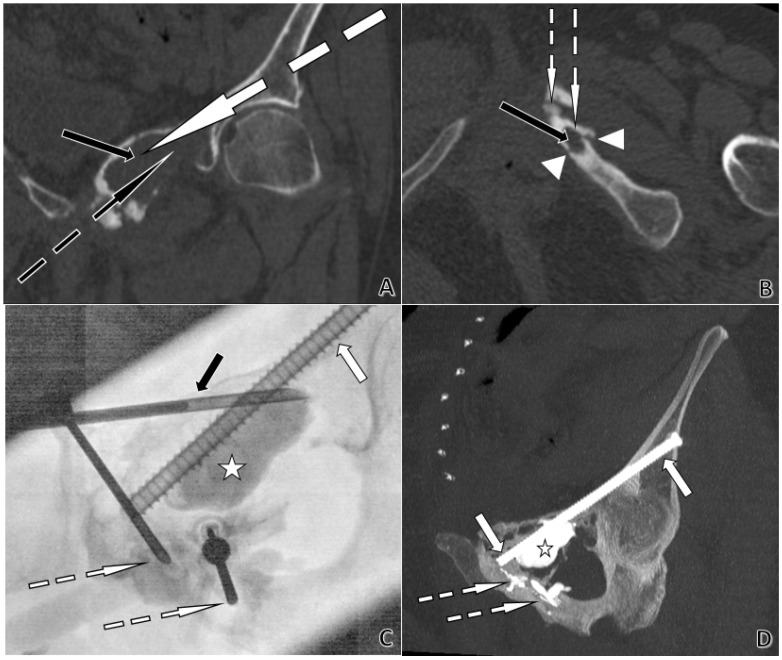
CT- and fluoroscopy-guided acetabular cementoplasty with screw fixation in a 74-year-old woman. Plasmacytoma extending from the left ischiopubic ramus to the pubic symphysis and the left acetabular roof, with cortical lysis and fractures of the ischiopubic ramus. (**A**)**. Coronal CT planning**. The black arrow indicates a large lytic lesion of the left iliopubic ramus. The dotted white arrow shows the planned trajectory for an 8-gauge trocar, permitting subsequent placement of a Kirschner-wire and then a 6.5 mm screw from the left iliac wing across the left acetabular roof toward the pubic symphysis. The dotted black arrow marks the planned trajectory for an 11-gauge trocar to cement the large lytic cavity. (**B**)**. Axial CT planning**. The black arrow shows a lytic lesion of the left ischiopubic ramus. Arrowheads delineate cortical lysis adjacent to the lesion. The dotted white arrows depict short, planned trajectories for two 13-gauge trocars, with only a thin cortex to cross, allowing the use of 13 G instrumentation. (**C**)**. Anteroposterior fluoroscopy during cement injection.** The white arrow identifies the 6.5 mm anterior-column screw. The dotted white arrows indicate the 13 G trocar positions used for cement injection. The black arrow points to the 11 G trocar. The star marks the large lytic cavity being filled with cement. (**D**)**. Post-procedure coronal CT with Maximum Intensity Projection (MIP) at completion**. The white arrows show the 6.5 mm screw extending from the left iliac wing across the left acetabular roof toward the pubic symphysis. The dotted white arrow highlights PMMA cement sealing osseous fissures. The star denotes the large lytic cavity filled with cement.

The clinical images in Figure 5 were obtained as part of routine clinical care. All images were fully anonymized prior to use. Written informed consent for publication was obtained from the patient. According to institutional policy, no additional IRB approval was required for retrospective anonymized educational material.

6. Funding and conflicts of interest

This study received support from the grant referenced in the manuscript, which has now been explicitly incorporated into the Funding statement.

## 3. Results

This section describes the factors influencing flow rate. The recommendations are based solely on theoretical considerations derived from Poiseuille’s equation and on conceptual clinical illustrations. No experimental injectability testing was performed. Accordingly, the flow relationships described should not be interpreted as quantitative or predictive.

### 3.1. Trocar Width (Internal Radius r)

The flow rate Q is proportional to the fourth power of the tube radius r. This means that the flow rate increases sharply as the radius increases. These considerations clearly demonstrate how trocar size drastically affects the flow rate in accordance with Poiseuille’s law. With all other factors being equal (including trocar length), switching from a 13-gauge to an 11-gauge trocar increases the flow rate by approximately a factor of 3.7, while using an 8-gauge increases it by about 7.5 (Table 1). An example of a trocar range, with different diameters and lengths, is shown in Figure 3. Conversely, very thin needles such as 18-gauge reduce the theoretical flow to a negligible fraction, limiting their use to highly specific situations (for example, cementoplasty of small and accessible pelvic fractures). In these cases, injection is only feasible with a very low-viscosity cement and within a very narrow time window, typically no more than 2–3 min after mixing, before viscosity rises and injection becomes impossible. Although Poiseuille’s equation cannot predict cement flow, the r^4^ relationship provides a qualitative heuristic illustrating why larger-gauge trocars tend to facilitate injection, even though actual cement flow is primarily governed by evolving viscosity and the complex geometry of the lesion.

In practice, clinicians should consider these principles when making compromises. For example, although introducing a larger trocar makes cement injection much easier and allows for the possibility of injecting a more viscous cement, it nonetheless requires a larger incision, potentially exposing the patient to greater risk and discomfort if cementoplasty is performed under local anesthesia.

### 3.2. Trocar Length «L»

The flow rate is inversely proportional to tube length. A longer tube imposes more resistance on the fluid, which proportionally reduces the flow rate. This contrasts with adjustments to the radius, which occur to the fourth power (r^4^, quartic dependence). This means that a minimal-length trocar should be used to reach the target. For example, if the target is 8 cm away, there is no point in using a 15 cm trocar. In this case, the shortest possible trocar should be used, and a 10 cm trocar should be chosen (Figure 3). Choosing a 15 cm trocar over a 10 cm one means unnecessarily reducing the flow rate by 33%, assuming all other parameters are the same. This underlines the importance of preparation for the procedure, location imaging and trocar planning. Additionally, at the end of the procedure, it will be necessary to purge the entire length of the trocar, which becomes more difficult to manage with a longer trocar.

### 3.3. Cement Selection and Changes in Viscosity “η” During the Procedure

Bone cement is not strictly Newtonian: its viscosity is time-dependent and evolves throughout the procedure [11]. It exhibits non-Newtonian, thixotropic rheology—viscosity decreases under sustained shear and partially recovers at rest—superimposed on changes driven by polymerization kinetics, temperature, and handling conditions, making its flow highly dependent on the stage of mixing and injection. Although Poiseuille’s law assumes laminar flow, the extremely low velocities and high viscosities involved in cementoplasty suggest that flow is likely to remain laminar; however, because cement is non-Newtonian and polymerizing, Reynolds number calculations cannot validate this assumption under clinical conditions. This confirms that Poiseuille’s law cannot be applied in any predictive, quantitative, or procedural manner to bone cement; at most, it provides a qualitative analogy for understanding how viscosity influences resistance to flow, without enabling anticipation or modeling of actual injection behavior. In addition, polymerization can be significantly altered by factors such as blood contamination or deviations from the recommended powder-to-liquid ratio, which may modify viscosity, working time, and mechanical properties during injection [12,13].

Several types of cement are available for clinical use in cementoplasty. Polymethylmethacrylate (PMMA) is by far the most widely used, due to its favorable balance between injectability, mechanical strength, and radiopacity. Alternatives such as calcium phosphate or bioactive cement are being developed, offering biological advantages including osteoconductivity, osteointegration, and the potential for gradual remodeling into native bone. However, their rheological behavior remains more restrictive for percutaneous use: these cements generally exhibit higher initial viscosity, shorter working times, and reduced injectability compared with PMMA, making them more difficult to deliver through narrow trocars or long access routes. Their setting time is also more sensitive to temperature and mixing consistency, which can limit their suitability in vertebral and metastatic indications where controlled, high-pressure injection is required [14].

PMMA cements are supplied in two components: a polymer powder (PMMA) and a liquid monomer (methyl methacrylate, MMA). PMMA comes in powder form, while MMA is the liquid monomer used to mix it with the PMMA powder [15]. When combined, they undergo an exothermic polymerization reaction, producing a paste that progressively hardens. By adjusting the powder-to-liquid ratio, the operator can modulate the viscosity, injection characteristics, and working time of the cement. A lower powder ratio reduces viscosity and prolongs injectability, but increases the risk of leakage into undesirable structures such as the epidural space, venous system, cardiac chambers, pulmonary arteries, soft tissues, or joints [8,16]. Conversely, a higher powder ratio increases viscosity, shortens working time, and reduces leakage risk, but requires higher injection forces and larger-gauge trocars. These properties make PMMA a versatile yet demanding material, requiring careful adaptation of viscosity to both lesion type and procedural goals.

Thus, cement viscosity varies according to the powder/liquid ratio, enabling the mixture to be adjusted to obtain a consistency suitable for injection.

Reducing cement viscosity can allow for the use of needles instead of trocars, e.g., 18 gauges, to approach a bone fissure without transosseous passage (e.g., fractured ischio-pubic ramus). On the other hand, this brings a greater risk of leakage into undesirable areas [8,16]. Reducing the amount of powder also increases the setting time, providing radiologists with more time to inject the cement. Conversely, it may be advisable to use high-viscosity cement or a higher quantity of powder in sites where the risk of leakage is very high or where the injection time required is short, e.g., cementoplasty of a single vertebra. Figure 4 shows how, as a function of time, the viscosity of 3 different cements will vary in different ways, with all other parameters being equal. The viscosity of the cement will increase during the procedure, thus reducing the flow rate at equal pressures. The increase in viscosity during the procedure occurs more rapidly when the temperature is higher. This explains why cement injected into the patient hardens more quickly than when injected at room temperature. Additionally, the cement hardens at different speeds depending on the injection site; a cementoplasty of a peripheral bone, where the skeleton is likely lower than core body temperature, will harden more slowly than in an L2 vertebra close to the retroperitoneum and abdominal organs temperature. It is important to avoid handling the syringes and injectors to prevent warming the cement prior to the procedure, which would increase its viscosity. Additionally, the cement and its liquid monomer can be pre-cooled in a refrigerator to lower their initial viscosity, thereby enhancing their injectability and extending the working time for precise application during the procedure.

### 3.4. Injection Pressure (P1)

Poiseuille’s law tells us that the flow rate is proportional to the difference in injection pressure and therefore depends on the pressure exerted at the trocar inlet. There are different ways of injecting cement, which influence the injection pressure. Small pre-filled syringes (1 mL) allow for more pressure than a larger syringe and can be connected directly to the trocar. Alternatively, injectors or extenders that are initially pre-filled with cement can be connected to the trocar, which provides more pressure than with pre-filled syringes. This also reduces radiation exposure for radiologists. In addition, “pushers” or flat-ended mandrels can be used to push cement into the trocar. For example, when the cement becomes very viscous at the end of the procedure, it may be necessary to insert the pushers to give more force and empty the trocar before removing it. This also has the advantage of avoiding cement leaks into the soft tissue when the trocar is removed. The choice of injection method used is often associated with the choice of cement. Companies generally require their cement to be used with their injection system. The choice of injection method should be considered in advance according to the case and take into account the following: number of sites, bone condition, and whether or not it is associated with an osteosynthesis procedure. Furthermore, during the procedure, an increase in resistance downstream from the trocar and in viscosity can occur quite suddenly, which needs to be compensated for during the injection by interventional radiologists.

### 3.5. Pressure Variation Downstream of the Trocar (P2)

Downstream pressure (“resistance”) will depend on the site to be treated. In the case of highly lytic or necrotic lesions, tumoral lesions offer little resistance to injections. In addition, kyphoplasty or thermal ablation prior to cementoplasty creates a cavity before cementing, which reduces downstream resistance. Resistance is greater in cases of mixed lesions. Resistance also depends on the state of the underlying bone, and the presence of porotic bone pre-existing the tumor lesion, if there is a tumor. Downstream pressure varies over time. The injection of cement into the site to be treated and the progressive filling of the bone downstream from the trocar will increase the resistance to injection over the course of the procedure.

### 3.6. Beyond Poiseuille: Device and Procedural Determinants

Factors in addition to the components of Poiseuille’s law play a part in equipment choices. This may include the type of tip and motorization of the trocar, prior thermal ablation and size of the trocar base, addition of cemented pin and associated osteosynthesis, and antibiotics in the cement. Furthermore, other factors influence cement distribution and the effectiveness of cementoplasty, as the position of the cement injected into the bone structure (e.g., cortical distance) [17].

### 3.7. Compromises and Personalized Medicine

Cementoplasty is not a standardized or formula-driven procedure, but rather a continuous balance of clinical and technical compromises. The parameters derived from Poiseuille’s law are not predictive tools and do not guide real-time procedural adjustments. They function solely as qualitative, conceptual heuristics, whereas procedural decisions rely on lesion morphology, cortical integrity, venous anatomy, material constraints, and the dynamic rheology of bone cement. Lesion type remains the first determinant: osteoporotic fractures, cystic or necrotic cavities, primary bone tumors, or metastatic involvement (lytic, mixed, or sclerotic) each require a specific strategy. Beyond pathology, the operator must integrate lesion size, cortical integrity, and the intended therapeutic objective—from stabilizing a fracture line to achieving complete cavity filling. The presence of osteosynthesis material (screws, spindles) may require synergistic reinforcement, influencing both cement viscosity and delivery approach [18,19]. Figure 5 illustrates this principle: in the same patient, trocars of different calibers and lengths were selected based on anatomical corridors, the depth of cortical bone, fracture configuration, and the need for additional fixation. Patient factors are equally decisive: age, frailty, comorbidities, and the possibility of local rather than general anesthesia all shape the strategy. Poiseuille’s law is presented in this manuscript solely as a conceptual and educational framework; it has no predictive, quantitative, or procedural validity for non-Newtonian bone cements and cannot model or anticipate cement flow, distribution, or safety during cementoplasty. Cementoplasty, therefore, exemplifies personalized medicine—not through strict physical modeling, but through the informed integration of physical principles, lesion biology, and patient-centered decision-making aimed at maximizing safety, function, and quality of life [20]. This approach exemplifies the essence of personalized medicine in interventional radiology, where safety and efficacy are maximized by tailoring the procedure to both patient and pathology.

## 4. Discussion

Although Poiseuille’s law cannot be applied in a predictive or quantitative manner to bone cement—given its non-Newtonian, shear-thinning behavior and the complex architecture of trabecular bone—it may serve as a conceptual educational tool for illustrating how certain physical parameters are theoretically related to resistance to flow. The exponential influence of radius, the proportional effect of length, and the general role of viscosity provide a simplified didactic framework that helps explain why resistance may vary under idealized conditions. This framework is intended solely to support conceptual understanding and does not describe, model, or predict actual cement behavior during injection.

In clinical reality, cement propagation is governed primarily by factors that lie outside the assumptions of Poiseuille’s law, including rapidly evolving viscosity during polymerization, temperature effects, irregular trabecular geometry, cortical defects, venous drainage pathways, and cement–bone interfacial interactions. These determinants explain why cement distribution is often unpredictable and why real-world procedural decisions cannot be derived from theoretical flow relationships. Accordingly, Poiseuille-based concepts should not be interpreted as informing viscosity selection, injection pressure, leakage prevention, or real-time procedural adjustments.

The clinical example presented in this Technical Note is provided for illustrative purposes only. It demonstrates how complex anatomical and pathological factors shape procedural strategy in practice, independently of any physical flow model. Its role is not to validate or apply Poiseuille’s law, but to contextualize the limits of conceptual modeling when confronted with real clinical situations.

In summary, the use of Poiseuille’s law in this manuscript is strictly educational. It does not aim to guide clinical practice, procedural planning, or safety decisions. Rather, it offers a qualitative framework to organize and communicate general physical notions related to resistance and flow under idealized conditions, while emphasizing the substantial gap between such models and the realities of cementoplasty.

## 5. Conclusions

Bone cement is a non-Newtonian, time-dependent material for which Poiseuille’s law has no predictive, procedural, or clinical validity. In this Technical Note, Poiseuille’s law is used solely as an educational framework to illustrate, in a qualitative manner, how idealized physical parameters such as radius, length, viscosity, and pressure are theoretically related to resistance to flow. This conceptual approach does not model cement behavior, does not inform clinical decision-making, and does not imply any impact on procedural planning, safety, or outcomes. Its purpose is limited to supporting didactic understanding of basic physical relationships under simplified conditions, while emphasizing the substantial gap between theoretical flow models and the realities of cementoplasty.

## Figures and Tables

**Table 1:** Relationship between the relative flow rate, with other factors being equal, as a function of trocar width (gauge) and length (cm).

Trocar Width (Gauge)	Trocar Length (cm)	Internal Radius (mm)	Relative Flow Rate (r^4^/L) Compared to 13 G (15 cm)
8 G	10	1.19	11.19
8 G	15	1.19	7.46
11 G	10	1.00	5.58
11 G	15	1.00	3.72
13 G	10	0.72	1.50
13 G	15	0.72	1.00 *
18 G	9	0.41	0.18

According to Poiseuille’s law, the internal radius of the trocar has the most dramatic effect on flow rate, since it is raised to the fourth power (r^4^). Thus, for the same length, the flow rate obtained with a larger-caliber trocar increases exponentially: compared to a 13-gauge trocar (reference, 15 cm), it is about 3.7 times higher with an 11-gauge trocar and approximately 7.5 times higher with an 8-gauge trocar. Conversely, very thin needles such as 18-gauge reduce the theoretical flow to a negligible fraction, limiting their use to highly specific situations (for example, cementoplasty of small and accessible pelvic fractures). The length of the trocar also directly influences resistance: extending the trocar from 10 cm to 15 cm reduces flow by one third, all other parameters being equal. These comparisons illustrate conceptual trade-offs between injectability and anatomical constraints under idealized conditions. The 18 G needle shows an extremely low theoretical flow rate, which explains its restricted use in very specific clinical scenarios, such as targeting fine bone fissures or performing highly localized injections where minimal cement diffusion is required. Relative flow rate computed as r^4^/L with 13 G-15 cm as reference* = 1.00; values rounded to two decimal places. Internal radius values were obtained from manufacturer specifications. The “relative flow rate” represents a theoretical calculation derived from the r^4^/L term of Poiseuille’s equation and does not correspond to experimental or measured flow rates.

## Data Availability

No new data were created or analyzed in this study.

## Abbreviations

The following abbreviations are used in this manuscript:

ΔP	Pressure difference (Pa)
L	Length (m)
MMA	Methyl methacrylate
Pa	Pascal (unit of pressure)
Pa.s	Pascal-second (unit of dynamic viscosity)
PMMA	PolyMethylMethAcrylate
Q	Volume flow rate (m^3^/s)
r	Radius (m)
η	Dynamic viscosity (Pa·s)
